# The Association between Periodontitis and Human Colorectal Cancer: Genetic and Pathogenic Linkage

**DOI:** 10.3390/life10090211

**Published:** 2020-09-18

**Authors:** Federica Di Spirito, Paolo Toti, Vincenzo Pilone, Francesco Carinci, Dorina Lauritano, Ludovico Sbordone

**Affiliations:** 1 Department of Medicine, Surgery and Dentistry “Schola Medica Salernitana”, University of Salerno, Via S. Allende, 84081 Baronissi (Salerno), Italy; capello.totipaolo@tiscali.it (P.T.); vpilone@unisa.it (V.P.); lsbordone@unisa.it (L.S.); 2 Complex Operating Unit of Odontostomatology, Head and Neck Clinical Department, Azienda Ospedaliero-Universitaria San Giovanni di Dio e Ruggi d’Aragona, 84121 Salerno, Italy; 3 Private Practice, Via Provinciale 87B, 55041 Camaiore (Lucca), Italy; 4 Department of Surgery, University of Pisa, Via Paradisa 2, 56124 Pisa, Italy; 5 Complex Operating Unit of General Surgery, Azienda Ospedaliero-Universitaria San Giovanni di Dio e Ruggi d’Aragona, 84121 Salerno, Italy; 6 Department of Morphology, Surgery and Experimental Medicine, University of Ferrara, Via Luigi Borsari 46, 44121 Ferrara, Italy; francesco.carinci@unife.it; 7 Department of Medicine and Surgery, Centre of Neuroscience of Milan, University of Milano-Bicocca, 20126 Milan, Italy; dorina.lauritano@unimib.it

**Keywords:** periodontitis, colorectal cancer, bioinformatics

## Abstract

Periodontitis has been associated with an increased risk of and mortality associated with human colorectal cancer (CRC). Current evidence attributes such an association to the direct and indirect effects of virulence factors belonging to periodontal pathogens, to inflammatory mediators and to genetic factors. The aims of the study were to assess the existence of a genetic linkage between periodontitis and human CRC, to identify genes considered predominant in such a linkage, thus named leader genes, and to determine pathogenic mechanisms related to the products of leader genes. Genes linking periodontitis and CRC were identified and classified in order of predominance, through an experimental investigation, performed via computer simulation, employing the leader gene approach. Pathogenic mechanisms relating to leader genes were determined through cross-search databases. Of the 83 genes linking periodontitis and CRC, 12 were classified as leader genes and were pathogenically implicated in cell cycle regulation and in the immune-inflammatory response. The current results, obtained via computer simulation and requiring further validation, support the existence of a genetic linkage between periodontitis and CRC. Cell cycle dysregulation and the alteration of the immuno-inflammatory response constitute the pathogenic mechanisms related to the products of leader genes.

## 1. Introduction

Periodontitis, as defined by Tonetti et al. and by Lang et al., is a “multifactorial microbially-associated inflammatory disease” affecting tooth-supporting structures and, ultimately, leading to tooth loss [1,2,3].

In the last decade, a growing body of evidence has reported the association between periodontitis and a variety of systemic inflammatory conditions and diseases, including atherosclerosis, diabetes, rheumatoid arthritis, and inflammatory bowel disease (IBD) [4,5,6]. Most notably, recent findings have also associated periodontitis with solid cancers, such as malignant neoplasms of the prostate, breast, lung, pancreas, and kidney [6,7]. Moreover, periodontitis has been associated with an increased risk of colorectal adenoma and colorectal cancer (CRC) development [8,9] and to an increased mortality from CRC [10].

Human colorectal cancer accounts for approximately ten percent of new cancer cases worldwide in males and 9.2% in females [11]. Considering the high mortality rate of CRC (eight percent and nine percent of cases, corresponding to 700,000 estimated deaths/year) [11], together with the associated morbidity, progress in treatment customization [12] and, above all, in primary and secondary prevention, indicates the importance of new insights into CRC etiopathogenesis [13].

Several environmental factors [12] involved in CRC carcinogenesis have been identified: unhealthy behaviors, such as consumption of red meat and alcohol, smoking, reduced physical activity, IBD [14] (comprising Crohn’s disease and ulcerative colitis) [15], and certain diseases and conditions, such as type 2 diabetes and obesity, which are related to systemic inflammation [16,17]. Indeed, it has been suggested that systemic inflammation may be critical to the development of CRC 13,16, and may link CRC with obesity, IBD, and periodontitis [6,7,18]. In particular, inflammatory mediators, which increase locally and systemically in periodontitis [10,19], together with carcinogens (i.e., nitrosamines), as well as microbial-associated virulence factors from periodontal pathogens, may underlie the association between human CRC and periodontitis.

In addition to environmental factors, genetic susceptibility and/or family history [12] have been recognized as important in ten percent of human CRC cases. The role of genetic factors [20] has been also demonstrated in periodontitis. Therefore, a genetic linkage between periodontitis and CRC has been hypothesized and was investigated in this study.

The primary aim of the present study was to assess, through an experimental investigation performed via computer simulation, the genetic linkages between periodontitis and human colorectal cancer, identifying all the genes involved in such an association, ranking them into cluster in descending order of relevance in such an association, and, finally, pointing out those genes presumed to be “leader” in the association between these disorders. Leader genes, which are considered to be predominant in the genetic determination of complex multi-factorial disorders, or in the genetic linkage between two disorders, as in the association between periodontitis and CRC, may reveal molecular targets for further investigations and focused therapies [20,21].

The secondary aim of the study was to characterize, through a review of current scientific evidence, the main function of leader gene products, their involvement in biological processes, and their role in the onset and progression of CRC and periodontitis, and to determine the putative pathogenic mechanisms associating periodontitis and CRC. Those preliminary data may highlight the possible clinical implications of the genetic linkages between periodontitis and human colorectal cancer and pave the way for targeted molecular experimentations [20,21].

## 2. Experimental Section

The present experimental study, being performed on computer, did not require either ethical approval or informed consent and was concluded on the 3 April 2019.

### 2.1. Analysis of the Genetic Linkage between Periodontitis and Human Colorectal Cancer (CRC)

A bioinformatic method, called leader gene approach [20], was employed to identify genes potentially involved in the association between periodontitis and CRC and especially those presumed to be predominant or “leader” in the genetic linkage between the two disorders.

**Figure 1:**
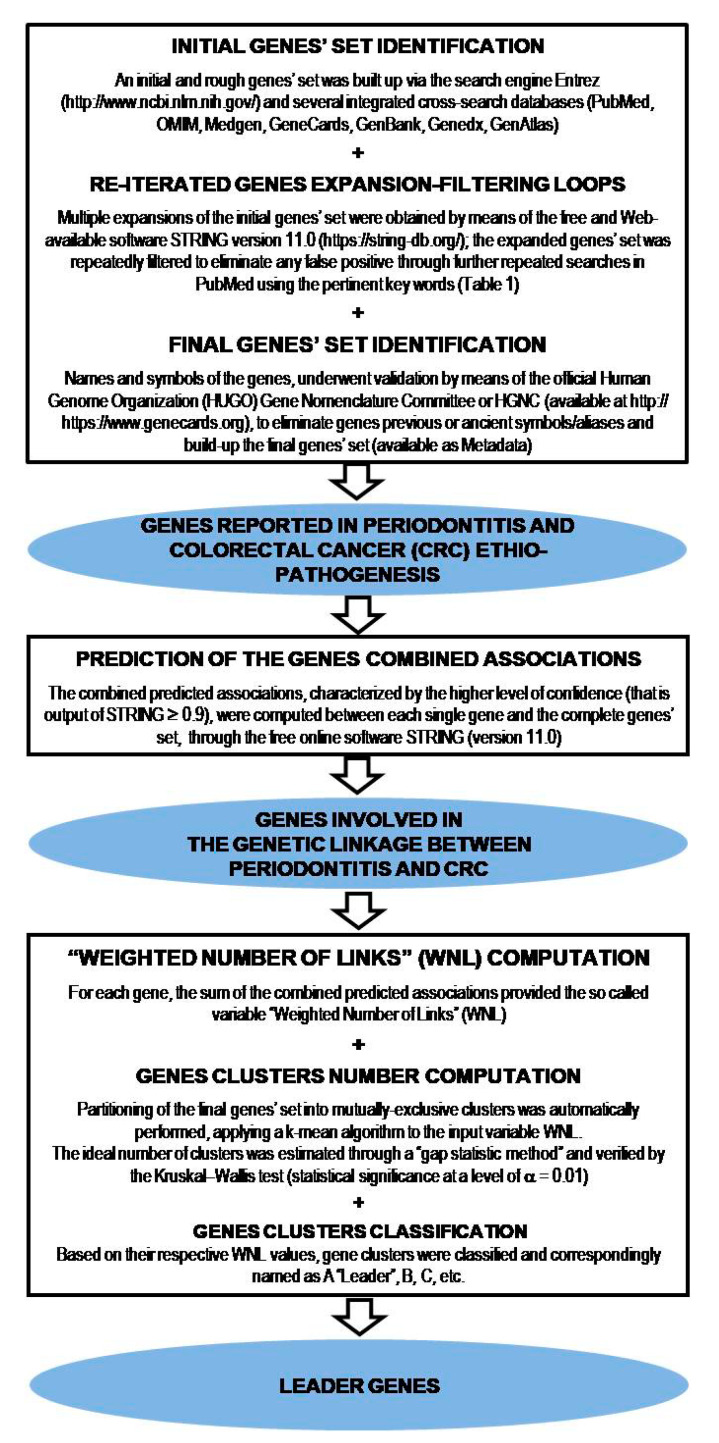
Step by step description of the gene clustering analysis procedure, performed via computer simulation, to investigate the existence of a genetic linkage between periodontitis and human colorectal cancer, and to identify leader genes.

The multi-step procedure, requiring freely available databases and a specific software program for each of the steps involved, is detailed in Figure 1 and summarized below.

Preliminarily, an initial set of genes involved in the above-mentioned phenomenon was built up through various integrated cross-search databases (PubMed, OMIM, Medgen, GeneCards, GenBank, Genedx, GenAtlas) using the search engine Entrez (http://www.ncbi.nlm.nih.gov/).

Repeated genes expansions, obtained through the web-available software STRING version 11.0 (https://string-db.org/) ELIXIR infrastructure, Hinxton, UK, and subsequent expanded genes filtrations to eliminate any false positive, through a further search with PubMed, all together defined as “expansion-filtering loops”, were performed.

The following key words, achieved by studies investigating either colorectal cancer or periodontitis or both of them [1,2,3,4,5,6,7,8,9,10,11,12,13,14,15,16,17,18,19,20], were employed in the literature search and were logically combined with the Boolean operators AND, OR, NOT.

The name as well as the symbol of each gene, derived by the above-mentioned databases, underwent validation by means of the official Human Genome Organization (HUGO) Gene Nomenclature Committee, or HGNC (available at https://www.genecards.org), in order to eliminate previous symbols or aliases.

The combined predicted associations, characterized by the higher level of confidence (that is a result with a score ≥ 0.9), were computed between each single gene and the complete gene dataset, through the free online software STRING (Version 11.0) [22]. The sum of these combined predicted associations scores provided the so-called the “Weighted Number of Links” (WNL) for each gene.

Automatic computations were performed on the whole data related to the genes included in the study. A k-mean algorithm was applied to the input variable WNL, and a partitioning of the overall dataset of genes into mutually-exclusive clusters was automatically performed and a “gap statistic method” was used to estimate the ideal number of clusters for the clusters from 2 to 12, as reported in Figure 1. Significant differences among WNLs of cluster groups obtained by the gap statistic method, were found by the Kruskal-Wallis test (statistical significance at a level of α = 0.01), verifying the accurate estimate of the number of clusters.

The resulting gene clusters were classified and correspondingly named as A, B, C, etc., based on their respective value of WNL centroid. The first genes cluster was identified as a ”leader” genes cluster, hypothesizing their possible central role in the phenomenon; in contrast, the last genes cluster, identified as ”orphans” genes, included genes without identified predicted associations (WNL = 0).

### 2.2. Determination of the Putative Pathogenic Mechanisms Associating Periodontitis and CRC

Leader genes characterization was performed, via the free online software STRING (Version 11.0) [22], to assess the main function of leader gene products and their involvement in biological processes. A further literature search, using the keywords reported in Table 1, was conducted on PubMed/MEDLINE and ScienceDirect search engines (using the same key words reported in Table 1), to investigate the role of leader genes in the onset and in the progression of CRC as well as of periodontitis and to highlight their putative pathogenic mechanisms in the genetic linkage between periodontitis and CRC.

## 3. Results

### 3.1. Analysis of the Genetic Linkage between Periodontitis and Human Colorectal Cancer

**Figure 2:**
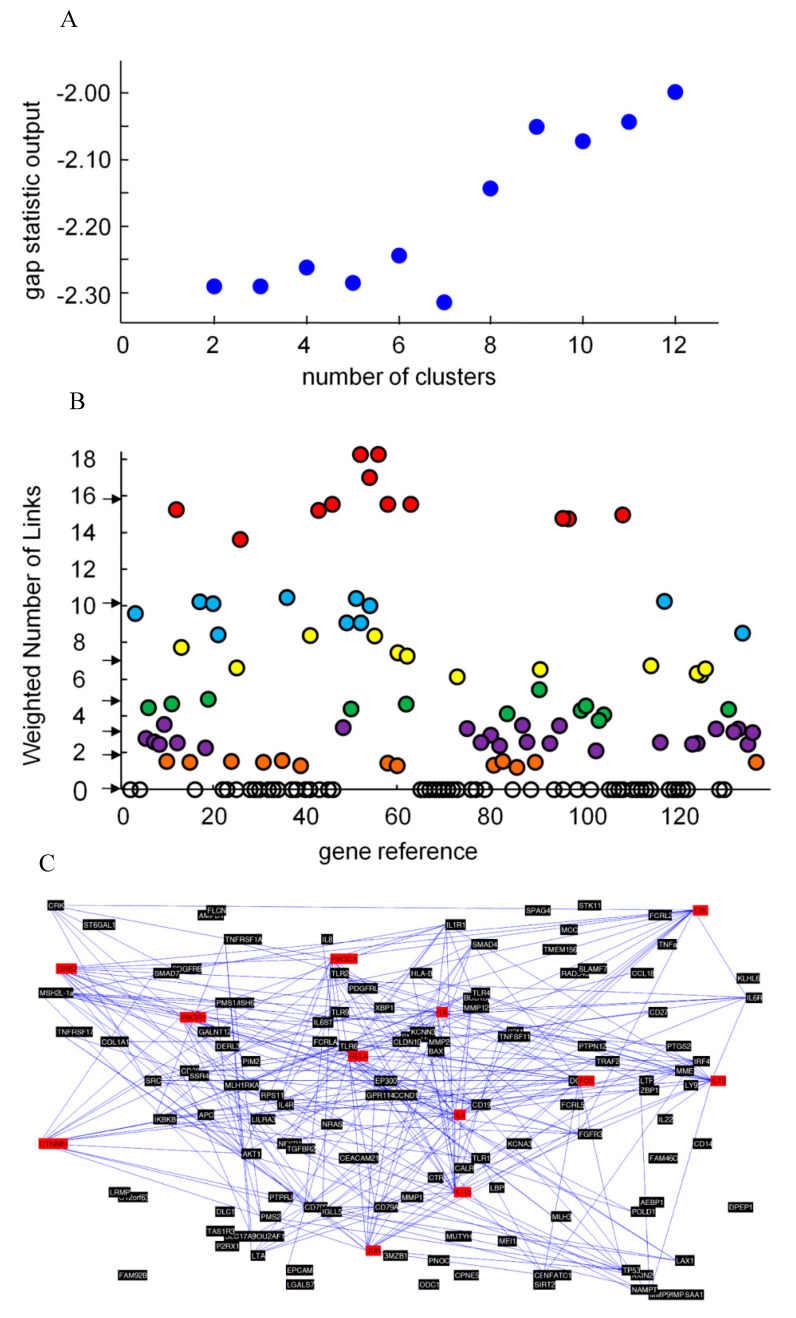
**A**–**C**. Data analysis for colorectal cancer and periodontal disease: (**A**) plot of the gap statistic method for estimating the number of clusters; (**B**) WNL for genes involved in the phenomenon. Black arrows are the centroids of the cluster groups: leader genes (in red); cluster B genes (in light blue); cluster C genes (in yellow); cluster D genes (in green); cluster E genes (in purple); cluster F genes (in orange); and ‘orphan’ genes (in clear); (**C**) final map of interactions of 137 genes involved in the genetic linkage between periodontitis and CRC according to STRING: leader genes are red; the lines that connect single genes represent predicted functional associations among proteins in the confidence view.

The final set of genes was composed of 137 genes. A complete description of the identified genes, including acronyms, identification numbers, validated names, cluster assignment, and their involvement in biological processes, is shown in Table A1. In compliance with the estimated optimal number of clusters, shown in Figure 2A, the 137 identified genes were divided into 7 clusters, designated as A, B, C, D, E, F, and orphan genes clusters. 

**Figure 3:**
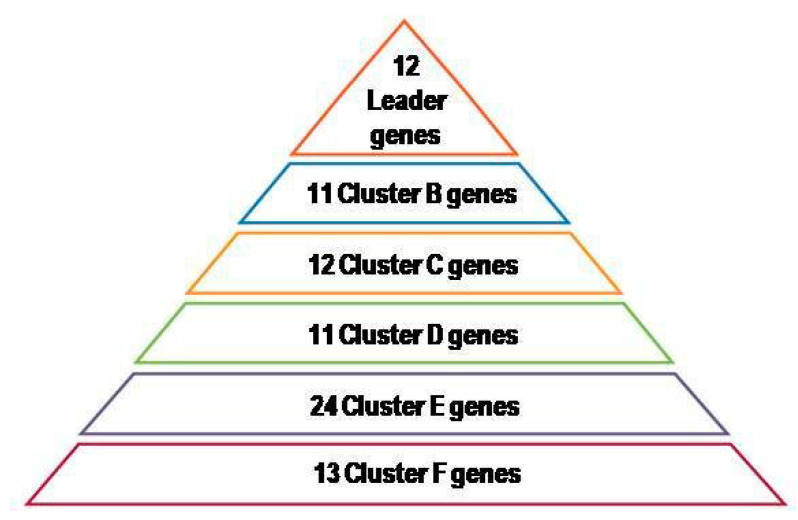
Gene classification in the seven clusters, designated from A to F, based on the number of predicted interactions of genes, excluding the last orphan genes cluster with no predicted interactions.

WNL computation is reported in Figure 2B. Depending on the WNL score, 54 genes, lacking combined predicted interactions (WNL = 0), were assumed not to be involved in the genetic linkage between periodontitis and CRC, and were, consequently, designated as orphan genes and excluded from the study; the remaining 83 genes, showing a WNL > 0 and the combined predicted interactions mapped in Figure 2C, were hierarchically grouped in descending order of WNL to the six clusters named from A to F, as illustrated in Figure 3.

In particular, the 12 genes, belonging to cluster A and defined as leader genes, were: E3 ubiquitin-protein ligase (CBL), catenin beta-1 (CTNNB1), proto-oncogene c-Fos (FOS), growth factor receptor-bound protein 2 (GRB2), interleukins 1B, 4, 6, 10 (IL1B, IL4, IL6, IL10), transcription factor AP-1 (JUN), phosphatidylinositol 4,5-bisphosphate 3-kinase catalytic subunit alpha isoform (PIK3CA), phosphatidylinositol 3-kinase regulatory subunit alpha (PIK3R1), and RELA proto-Oncogene NFKB subunit or transcription factor p65 (RELA).

CBL encodes for an enzyme targeting substrates for proteasomal degradation.

CTNNB1 encodes for β-catenin, a subunit of the adherens junctions complex, regulating cell growth and adhesion and Wnt responsive genes (i.e., c-Myc) expression, leading to cell cycle progression.

FOS is an oncogene encoding for the c-Fos protein, which heterodimerizes with c-Jun, encoded by JUN (Transcription factor AP-1), to form the transcription factor AP-1, involved in cell proliferation, differentiation, apoptosis, and cancerous transformation.

GRB2 gene encodes for a protein binding the epidermal growth factor (EGF) receptor, activating several signaling pathways.

IL1B, IL4, IL6, IL10 are active in immune-regulation and inflammation, as discussed below. 

PIK3CA and PIK3R1 are centrally involved in several cancers.

RELA (p65), along with NFKB1 (p50), make-up the NFKB complex, which regulates the transcription of several genes encoding for pro-inflammatory cytokines https://www.genecards.org) [22].

### 3.2. Determination of the Putative Pathogenic Mechanisms Associating Periodontitis and CRC

The characterization of the 12 leader genes in the genetic linkage between periodontitis and human colorectal cancer is reported in Table 2. Identified leader genes were involved in cell signaling (i.e., CTNNB1, CBL, GRB2, PIK3CA, PIK3R1), transcriptional pathways (i.e., JUN, RELA), cell proliferation/differentiation (i.e., FOS), and immuno-inflammatory processes (i.e., IL1B, IL4, IL6, IL10). Current evidence of the role of leader genes in CRC and in periodontitis onset and progression, as well as the putative pathogenic mechanisms is reported in Table 2.

## 4. Discussion

Periodontitis and human colorectal cancer are complex multi-factorial disorders, dealing with a multitude of genes, which are interconnected by several heterogeneous networks, and whose products are involved in a wide range of biological pathways [20]. In view of this fact, the present experimental investigation of the genetic linkages between periodontitis and CRC was conducted through a bioinformatic method, called “leader gene approach” [20]. This multi-step procedure, as described above, is especially useful in identifying the highest priority genes in the investigated phenomenon [20,21] and provides the necessary synthesis and analysis of the overwhelming amount of raw bioinformatic data generated. Ranking genes hierarchically and identifying leader genes consistently revealed those genes, and their related products, which are mainly involved in the genetic linkage between periodontitis and CRC (Table 2). Such bioinformatic data were subsequently integrated with current evidence to reveal cellular functions and biological processes carried out by the gene products, and were interpreted in view of the available clinical and experimental findings to determine the putative pathogenic mechanisms associating periodontitis with CRC.

### 4.1. Genetic Linkages between Periodontitis and Human Colorectal Cancer: Leader Genes and Putative Pathogenic Mechanisms

Among the 137 genes (complete final gene dataset available as metadata) reported in periodontitis and CRC ethio-pathogenesis, 83 were involved and 12 (“cluster A” or “leader” genes) were considered to play a predominant role in the genetic linkage between both disorders. Notably, four of the cluster A genes, specifically, CBL, GRB2, PIK3R1, and RELA, were also ranked among the five leader genes previously identified in periodontitis [20]. Nuclear factor kappa B p105 subunit (NFKB1), instead, which is considered as a leader gene in periodontitis, was assigned to cluster C in the present study. These results may support the existence of a possible genetic linkage between periodontitis and CRC.

The characterization of the currently identified leader genes, reported in Table 2, revealed their involvement in several biological processes, such as cell signaling (i.e., CTNNB1, CBL, GRB2, PIK3CA, PIK3R1), transcriptional pathways (i.e., JUN, RELA), cell proliferation/differentiation (i.e., FOS) and immuno-inflammatory processes (i.e., IL1B, IL4, IL6, IL10; see Table 2) [22]. Evidence supporting the role exerted by leader genes in both CRC and periodontitis pathogenesis [23,24,25,26,27,28,29,30,31,32,33,34,35,36,37,38,39,40,41], reported in Table 2, suggested that the pathogenic mechanisms underlying the association between periodontitis and CRC may be mainly related to the effect of the products of the leader genes on cell cycle dysregulation and on alteration of the immuno-inflammatory response. 

Leader genes acting in cell cycle regulation, such as CTNNB1, FOS, JUN, GRB2, PIK3CA, and PIK3R1, may affect homeostasis in both colonic cells and periodontal tissues, causing, if dysregulated, colonic cell proliferation and malignant transformation, on the one hand, and periodontitis development and progression, on the other, as described in Table 2 [22,23,24,25,26,27,28,29,30,31,32]. 

Leader genes affecting the immune-inflammatory response, such as IL1B, IL4, IL6, IL10, CBL, and RELA, may underlie a possible bi-directional relationship between the disorders, as described below [20,33,34,35,36,37,38,39,40]. Moreover, in addition to leader genes affecting the immune-inflammatory response, NFKB, which has been ranked among cluster C genes and is functionally related to RELA, regulates the transcription of several genes, also encoding for pro-inflammatory cytokines. NFKB is constitutively inactivated and its activation, with subsequent immuno-inflammatory response alteration, may be due to a dysregulation in the ubiquitin–proteasome system, which is a mechanism of intracellular protein degradation, occurring in atherogenesis, neurodegenerative and autoimmune diseases, and, possibly, in IBD and CRC [41,42]. Current knowledge about the role of the cellular ubiquitin–proteasome system dysregulation, and subsequent NFKB activation in periodontitis, is still limited, but it may explain the presence of the E3 ubiquitin-protein ligase (CBL) gene among the leader genes in the genetic linkage between periodontitis and CRC, although no evidence is available relating CBL to periodontitis [20].

### 4.2. Genetic Linkages between Periodontitis and Human Colorectal Cancer: Cytokines and Systemic Inflammation

Periodontal tissue destruction, occurring in periodontitis, is microbially initiated and sustained by the dysregulation of the immune-inflammatory processes [5]. A body of evidence has shown that cytokines produced in inflamed periodontal tissues, together with virulence factors from periodontal pathogens and oral microbial agents, may gain access to the circulation, and, consequently, induce systemic inflammation [5,43]. Accordingly, it has been proposed that non-resolving periodontal inflammation may affect systemic inflammatory diseases and that cytokines may be considered as a possible pathogenic link between periodontitis and various systemic diseases, including IBD and CRC [4,5,6,7,38,44].

It is well known that IBD has oral mucosal manifestations, such as pyostomatitis vegetans and aphthous stomatitis, and it has been reported that subjects suffering from Crohn’s disease show a higher risk of periodontitis compared to non-IBD subjects [8,45]. In addition, evidence suggests that periodontal pathogens, especially Fusobacterium nucleatum, may be involved in IBD [9,10] and colorectal adenomas [46], and that cytokines induced by periodontal pathogens and released in periodontitis may predispose to neoplastic transformation of chronic colitis, favoring colorectal carcinogenesis [12,46,47]. In more detail, oral fusobacterium nucleatum, which is abundant in the oral cavity and increased in periodontal pockets, is mobile and caplable to bind, through the Fusobacterium adhesin A (FadA), both to vascular endothelial-cadherins, gaining access to systemic circulation, and to (E)-cadherin on epithelial cells, stimulating the growth of tumor cells. Binding to (E)-cadherins on colorectal adenoma and cancer cells, FadA, which is only detectable on oral Fusobacterium species, activates the transcriptions of those oncogenes regulated by b-catenin, which is the product of the leader gene CTNNB1, and of some genes involved into the immune-inflammatory response, including IL6, which is presently ranked as a leader gene, and NFKB, belonging to cluster C genes [46]. From this standpoint, periodontitis may be considered as a possible risk factor for CRC genesis in IBD subjects, as it accounts for poor metabolic control in diabetic patients [48]. Such an inter-relationship may rely on the fact that both IBD and periodontitis share a multifactorial etiology, as well as the pathogenic mechanisms affecting the local immuno-inflammatory response, which leads to the genesis of a systemic inflammation [45]. Analogously, it may be supposed that those periodontal cytokines, which are listed among leader genes products, may enhance colonic tumor-associated inflammation, and may subsequently be considered as a risk factor for cancer progression in CRC subjects. As a counterpart, along with the tumor-associated inflammatory environment, CRC cells themselves release inflammatory mediators, which self-sustain neoplastic cell growth and enhance the cancerous cells’ interactions with the surrounding stroma and immune cells, favoring, in turn, CRC progression and invasion [13,49]. Since CRC inflammatory mediators have been identified as leader genes in the present study, it may be hypothesized, as previously proposed for cytokines released in diabetes [43], that CRC cytokines may negatively affect periodontitis onset and development, altering the immune-inflammatory response in periodontal tissues.

### 4.3. Genetic Linkages between Periodontitis and Human Colorectal Cancer: Possible Clinical Implications

The findings discussed, certainly requiring validation by larger studies, may provide preliminary data for further research, especially considering the beneficial clinical applications potentially offered by the insight into the mechanisms associating periodontitis and CRC. Indeed, if the results presented, which suggest a central role for cytokines and systemic inflammation in the genetic bi-directional linkage between periodontitis and CRC, are validated, periodontitis management may be included in CRC prevention and treatment plans. Complex multi-factorial disorders, such as periodontitis and CRC, significantly impact on the quality of life, present life-threatening risks, and imply a heavy burden on society. Therefore, highlighting the genetic traits of such disorders may pave the way for primary prevention strategies, which are essential to reduce the biological impact as well as the healthcare costs of these disorders. The improved understanding of the putative pathogenic mechanisms associating periodontitis with CRC may encourage a multidisciplinary approach, which is strongly advocated for such complex multifactorial disorders. 

From this standpoint, oral health professionals may also become part of CRC screening plans, introducing, in their daily practice, general health promotion and disease prevention goals, and including risk assessment for both oral and systemic diseases. CRC screening might be improved by the provision of broader dental health records, with the potential to identify subjects at risk for CRC development for referral to a physician. In addition, based on the definition of oral health as a component of general health affecting the quality of life [50], oral health professionals may widen their activity in an interprofessional setting, providing oral and periodontal evaluation and necessary treatments, in CRC subjects referred by other health professionals, integrating the patient’s medical care in therapeutic and follow-up plans.

Periodontal treatment may be proposed as a CRC primary prevention strategy, in subjects considered at higher risk for CRC development, such as those suffering from IBD, in order to decrease the systemic inflammation and the related pro-carcinogenic environment. However, threshold values of cytokines in inflamed periodontal tissues, capable of inducing systemic inflammation and subsequently increasing the risk for colorectal cancer genesis, in IBD subjects have not yet been defined. Furthermore, the quantitative assessment of periodontal cytokines is even more complicated than the qualitative one, since it may actually be biased by the accidental detection of inflammatory mediators possibly derived from mucosal inflammation and orally administered drugs, in whole saliva analysis, and by the need for full mouth sampling, in gingival crevicular fluid analysis [51]. For these reasons, identifying those IBD subjects potentially exposed to a higher risk of systemic inflammation induced by periodontitis, and of consequent malignant transformation of chronic colitis, may be impracticable. Thus, periodontitis prevention and treatment, which potentially reduces systemic inflammation and, consequently, decreases the risk for malignant transformation of chronic colitis, may be routinely included in all IBD subjects’ treatment plans. Moreover, periodontal treatment, reducing the periodontal microbial load and the related cytokine levels, may decrease the systemic spread of inflammatory mediators and of Fusobacterium nucleatum, specifically, presumed to be associated with CRC, beyond IBD, lesions [41,46], and to favor tumor-associated environment, and may, therefore, constitute a secondary and/or tertiary prevention strategy in subjects affected by CRC.

## 5. Conclusions

Four out of the five leader genes previously identified for periodontitis (CBL, GRB2, PIK3R1, and RELA) were also listed as leader genes in the investigated phenomenon, carefully supporting the genetic linkages between CRC and periodontitis, and suggesting the need for a multi-disciplinary approach, also involving oral health professionals, to CRC subject management. 

IL1B, IL4, IL6, IL10 were also ranked among leader genes, suggesting a central role for systemic inflammation in the genomic relationship between CRC and periodontitis; in particular, periodontitis may be linked to IBD, and, in turn, to CRC, both affecting the inflammatory pro-carcinogenic and tumor-associated environment and acting in an indirect way in the “inflammation-dysplasia” carcinogenic sequence, favoring colorectal cancer development. In this perspective, periodontitis management may be proposed as a CRC primary prevention strategy, especially in patients considered at higher risk for CRC development, such as IBD subjects. Indeed, periodontal therapy would reduce the periodontal microbial charge, and, consequently, the systemic widespread of bacterial toxins and of periodontal pathogens them-selves, including Porphyromonas gingivalis and Fusobacterium nucleatum, supposed to be associated with IBD and CRC lesions. Moreover, periodontal treatment and healthy periodontal conditions would indirectly decrease the systemic inflammation and the related CRC pro-carcinogenic environment, as a part of the CRC treatment strategy.

## Figures and Tables

**Table 1:** The following key words, achieved by studies investigating either colorectal cancer or periodontitis or both of them [16,18,19,21,23,24,25,26,27,28,29,30,31,32,33,34,35,36,37,38,39,40,41], were employed in the literature search and were logically combined with the boolean operators AND, OR, NOT.

	Key Words
(1)	gene AND human
(2)	cancer
(3)	carcinoma
(4)	2 OR 3
(5)	colon
(6)	colonic
(7)	rectal
(8)	CRC
(9)	5 OR 6 OR 7 OR 8
(10)	periodontitis
(11)	periodontal disease
(12)	periodontal inflammation
(13)	gingivitis
(14)	periodontal disruption
(15)	10 OR 11 OR 12 OR 13 OR 14
(16)	1 AND 4 AND 9 AND 15

**Table 2:** Description of the leader genes identified in the genetic linkage between periodontitis and human colorectal cancer: leader genes product(s) main function’, as per the free online software STRING (version 11.0) [22]; role in CRC development and progression; role in periodontitis onset and progression; putative pathogenic mechanisms related to the effects of the products of leader genes.

Leader Genes	Main Function	Role in CRC	Role in Periodontitis	Putative Pathogenic Mechanisms
CTNNB1	Cell signaling	Mutated in up to 90% of colonic tumors; responsible for initial tissue dysplastic transformation [22]; encodes for β-catenin, a subunit of the adherens junctions complex, regulating cell growth and adhesion and Wnt responsive genes (i.e., c-Myc) expression, leading to cell cycle progression.	Its product, β-catenin, is detectable in periodontal ligament cell nuclei in mice, potentially influencing periodontal ligament homeostasis [23]; regulates Wnt responsive genes. Wnt stimulus induces osteogenic lineage commitment [23], while Wnt depletion is involved in alveolar bone loss.	Cell cycle dysregulation
FOS	Gene(s) transcription, cell signaling, cell proliferation and differentiation	rs7101 and rs1063169 FOS single nucleotide polymorphisms are considered at higher risk of CRC onset [24] and its expression increases in CRC lesions [25]. In addition, a different member of the FOS family, named Fra-1, is over-expressed in colonic cancer cells, particularly in those acquiring motility and invasive ability [25]. Moreover, FOS may participate in the inflammatory microenvironment associated with CRC [25].	May be implicated in periodontitis development and progression through the interaction with prostaglandin-endoperoxide synthase 2, affecting the T-cell receptor (TCR) signaling [26].	Cell cycle dysregulation
JUN	Gene(s) transcription, cell signaling, cell proliferation, and differentiation inflammation	Its product, c-Jun, heterodimerizes with c-Fos protein, encoded by FOS, to form the transcription factor AP-1 (see above). Involved in cell proliferation, differentiation, apoptosis, and malignant transformation [24,27].	Its product, c-Jun, heterodimerizes with c-Fos protein, encoded by FOS, to form the transcription factor AP-1 (see above). Involved in cell proliferation, differentiation, apoptosis, and malignant transformation [24,27].	Cell cycle dysregulation
GRB2	Cell signaling	Its products stimulate colonic cell proliferation [28]; in particular, the Grb2-associated binding protein 2 (Gab2) has been found responsible for epithelial mesenchymal transition and consequent CRC metastasis development [29].	Its products bind to the epidermal growth factor (EGF) receptor. EGF signaling in the periodontal tissue, indirectly affected by GRB2 expression, is considered essential in tissue regeneration; thus, its interruption may affect healing and regeneration processes. Indeed, EGF ligand alterations, secondary to the effect of the peptidylarginine deiminase enzyme, released by porphyromonasgingivalis, interfere with EGF signaling, and, potentially, favor periodontitis progression [30].	Cell cycle dysregulation
PIK3CA	Cell proliferation, cell survival	The most frequently mutated gene in breast cancer and is centrally involved in other malignancies [22].	n.a.	Cell cycle dysregulation
PIK3R1	Cell signaling	Phosphorylated by PIK3CA, it is downregulated in CRC cells [31].	It is considered as a marker of severe periodontitis [32].	Cell cycle dysregulation
IL6	Inflammation	Induces CRC cell growth and invasion; and higher levels of IL6 have been detected in the serum from CRC patients compared to controls [33].	Stimulates osteoclastogenesis [34], has been found associated with chronic as well as aggressive periodontitis and, together with IL6R, IL6ST, IL4R, and IL1R1 may link periodontitis to other diseases [20].	Immuno-inflammatory response
IL1B	Immune response	In CRC cells it is produced in higher concentrations compared to healthy surrounding tissues, possibly activating the NFKB signaling pathway [35].	IL1-889 C/T gene polymorphism has been associated with severe periodontitis [34] and its role in periodontitis pathogenesis has long been advocated [36].	Immuno-inflammatory response
IL4	Immuno-inflammatory process	Produced by activated T helper 2 lymphocytes, may reduce cancer-directed response operated by the immune system, encouraging cancer invasion and metastasis. Through its binding to Type II IL-4 receptor α (IL-4Rα) and JAK/STAT signaling activation, it favors survival of cancer cells and immunosuppression, so that a dysregulation in IL-4 signaling or IL-4Rα gene polymorphisms may be associated with cancer, including CRC [37].	Plays a protective role in periodontitis progression, reducing alveolar bone loss. Consequently, IL4 gingivo-crevicular fluid levels are higher in periodontally healthy subjects and after non-surgical periodontal treatment. In addition, the IL4-590 C/T polymorphism has been reported as potentially associated with an increased risk of periodontitis development [38].	Immuno-inflammatory response
IL10	Gene(s) transcription	Its deficiency favors IBD malignant transformation to CRC [4,39], through the so called “inflammation-dysplasia-carcinoma sequence”, an alternative to the well-known “adenoma-carcinoma sequence” [2].	Anti-inflammatory cytokine, down-regulating monocyte-macrophage response. Its gene polymorphism has been associated with periodontitis development in Caucasians [34].	Immuno-inflammatory response
RELA	Cell signaling	Its expression is higher in malignant compared to healthy colonic cells, as well as in breast, liver, pancreatic, and gastric cancers, although its role in cancerogenesis, as well as in periodontitis, is still not fully elucidated [40].	It is also classified as leader gene in periodontitis probably because it is functionally related to NFKB pro-inflammatory activity [22].	Immuno-inflammatory response
CBL	Cell signaling	It may be related to inflammatory bowel disease (IBD) and CRC [41], as well as to atherogenesis, and neurodegenerative and autoimmune diseases, by a de-regulation in the ubiquitin–proteasome system, with subsequent NFKB activation and immuno-inflammatory response enhancement.	No evidence is available relating CBL to periodontitis [20].	Immuno-inflammatory response

## Appendix A

**Table A1:** Identified genes acronyms, identification numbers, validated names, cluster assignment, and biological process(es) involvement description, as per the free online software STRING (version 11.0) [22].

Gene Acronym	Gene Identification Number	Gene Official Name	Protein Main Function/Biological Process (Es) Involvement	Gene Cluster Assignment
CBL	12	E3 ubiquitin-protein ligase CBL	Cell signaling	A
CTNNB1	26	Catenin beta-1	Cell signaling	A
FOS	43	Proto-oncogene c-Fos	Gene (s) transcription, cell signaling, cell proliferation, and differentiation	A
GRB2	46	Epidermal Growth Factor Receptor-Binding Protein GRB2	Cell signaling	A
IL1B	52	Interleukin 1 beta	Inflammation	A
IL4	54	Interleukin 4	Immune response	A
IL6	56	Interleukin 6	Immuno-inflammatory process	A
IL10	58	Interleukin 10	Inflammation	A
JUN	63	Transcription factor AP-1	Gene(s) transcription	A
PIK3CA	96	Phosphatidylinositol 4,5-bisphosphate 3-kinase catalytic subunit alpha isoform	Cell proliferation, cell survival	A
PIK3R1	97	Phosphatidylinositol 3-kinase regulatory subunit alpha	Cell signaling	A
RELA	109	RELA Proto-Oncogene, NF-KB Subunit or Transcription factor p65	Sub-unit of the transcription factor NF-kappa-B	A
AKT1	2	RAC-alpha serine/threonine-protein kinase	Cell proliferation, cell survival, angiogenesis	B
CD19	16	B-lymphocyte antigen CD19	Immune response	B
CD79A	19	B-cell antigen receptor complex-associated protein alpha chain	Immune response	B
CD79B	20	B-cell antigen receptor complex-associated protein beta chain	Immune response	B
EP300	35	Histone acetyl transferase p300	Regulates genes transcription via chromatin remodeling	B
IGLL5	48	Immunoglobulin lambda like polypeptide 5	Associated with solitary osseous plasmacytoma	B
IKBKB	50	Inhibitor of nuclear factor kappa-B kinase subunit beta	Cell signaling (NF- kappa-B pathway)	B
IL-1a	51	Interleukin-1 alpha	Immuno-inflammatory process	B
IL1R1	53	Interleukin-1 receptor type 1	Cell signaling	B
SRC	117	Proto-oncogene tyrosine-protein kinase Src	Gene(s) transcription, immune response, cell cycle regulation, cell adhesion, and migration	B
TP53	134	Cellular tumor antigen p53	Cell cycle regulation	B
CCND1	13	G1/S-specific cyclin-D	Cell cycle regulation	C
CRK	25	Adapter molecule crk	Phagocytosis of apoptotic cells, cell motility	C
FGFR3	41	Fibroblast growth factor receptor 3	Cell proliferation, differentiation, and apoptosis, and skeleton development	C
IL4R	55	Interleukin-4 receptor subunit alpha	Immune response	C
IL6R	60	Interleukin-6 receptor subunit alpha	Immuno-inflammatory process	C
IRF4	62	Interferon regulatory factor 4	Immune response, dendritic cell differentiation	C
LTA	73	Lymphotoxin-alpha	Immune response	C
NFKB1	91	Nuclear factor NF-kappa-B p105 subunit	Cell signaling, immuno-inflammatory process, cell cycle regulation, and differentiation, tumorigenesis	C
SMAD4	115	Mothers against decapentaplegic homolog 4	Muscle physiology	C
TLR2	125	Toll-like receptor 2	Immune response	C
TLR4	126	Toll-like receptor 4	Immune response	C
TLR6	127	Toll-like receptor 6	Immune response	C
AURKA	5	Aurora kinase A	Cell cycle regulation	D
B2M	10	Beta-2-microglobulin	Immune response	D
CD38	18	ADP-ribosylcyclase/cyclic ADP-ribosehydrolase 1	Synthesizes the second messengers cyclic ADP-ribose and nicotinate-adenine dinucleotide phosphate	D
IGJ	49	Immunoglobulin J chain	Immune response	D
IL6ST	61	Interleukin-6 receptor subunit beta	Cell signaling, immune response, hematopoiesis, pain control, bone metabolism	D
MMP9	83	Matrix metalloproteinase-9	Extracellular matrix degradation, leukocyte migration, bone osteoclastic resorption	D
NFATC1	90	Nuclear factor of activated T-cells, cytoplasmic 1	Immuno-inflammatory process, osteoclastogenesis	D
PMS1	99	PMS1 protein homolog 1	DNA repair	D
PMS2	100	Mismatch repair endonuclease PMS2	DNA repair	D
POU2AF1	103	POU domain class 2-associating factor	Immune response	D
PTGS2	104	Prostaglandin G/H synthase 2	Inflammation	D
TNFRSF1A	131	Tumor necrosis factor receptor superfamily member 1A	(Pro) Apoptosis	D
APC	4	Adenomatous polyposis coli protein	Tumor suppressor (Wnt pathway)	E
AXIN2	6	Axin-2	Cell signaling (Wnt pathway)	E
BAX	7	Apoptosis regulator BAX	(Pro) Apoptosis	E
BMPR1A	8	Bone morphogenetic protein receptor type-1A	Chondrocyte differentiation, Adipogenesis	E
CALR	11	Calreticulin	Cell endoplasmic reticulum formation	E
CD27	17	CD27 antigen	Immune response	E
HLA-B	47	HLA class I histocompatibility antigen, B-7 alpha chain	Immune response	E
LTF	74	Lactotransferrin	Immuno-inflammatory process, protection against cancer development and metastasis	E
MLH1	77	DNA mismatch repair protein Mlh1	DNA repair	E
MME	79	Neprilysin	Opioid peptides, angiotensin-2, -1, -9 and atrial natriuretic factor degradation	E
MMP2	81	Matrix metalloproteinase-2	Inflammation, tissue repair, angiogenesis, tumor invasion	E
MSH2	86	DNA mismatch repair protein Msh2	DNA repair	E
MSH6	87	DNA mismatch repair protein Msh6	DNA repair	E
NRAS	96	GTPase NRas	Binds GDP/GTP and possesses intrinsic GTPase activity	E
PDGFRB	94	Platelet-derived growth factor receptor beta	Tyrosine-protein kinase acting as cell-surface receptor, playing an essential role in blood vessel development	E
POLD1	102	DNA Polymerase Delta 1 Catalytic Subunit	Plays a crucial role in high fidelity genome replication, requiring the presence of accessory proteins POLD2, POLD3, and POLD4 for full activity	E
SMAD7	116	Mothers against decapentaplegic homolog 7	TGF-beta inhibition	E
TGFBR2	123	TGF-beta receptor type-2	Cell cycle regulation (epithelial and hematopoietic cells), cell proliferation and differentiation (mesenchymal cells) Immune response	E
TLR1	124	Toll-like receptor 1	Immune response	E
TLR9	128	Toll-like receptor 9	Immune response	E
TNFRSF17	132	Tumor necrosis factor receptor superfamily member 17	Immune response	E
TNFSF11	133	Tumor necrosis factor ligand superfamily member 11	Immune response	E
TRAF2	135	TNF receptor-associated factor 2	NF-kappa-B and JNK activation, cell survival and apoptosis regulation, immune response	E
XBP1	136	X-box-binding protein 1	Cardiac, hepatic, and secretory tissue development	E
BUB1B	9	Mitotic checkpoint serine/threonine-protein kinase BUB1 beta	Cell cycle regulation	F
CCL18	14	C-C motif chemokine 18	Immune response	F
COL1A1	23	Collagen alpha-1 (I) chain	Member of group I collagen	F
DCC	30	Netrin receptor DCC	Nervous system development	F
EPCAM	34	EPCAM Epithelial cell adhesion molecule	Immune response	F
FCRLA	38	Fc receptor-like A	Immune response	F
IL8	57	Interleukin-8	Immune response	F
IL22	59	Interleukin-22	Inflammation	F
MMP1	80	Matrix metalloproteinase-1	Types I, II, III, VII, and X collagens degradation	F
MMP7	82	Matrix metalloproteinase-7	Casein, type I, III, IV, and V gelatins and fibronectin degradation	F
MZB1	85	Marginal zone B- and B1-cell-specific protein	Immune response	F
NAMPT	89	Nicotinamide phosphoribosyl transferase	Immune response, anti-diabetic function	F
ZBP1	137	Z-DNA-bindin gprotein 1	Immune response	F
AEBP1	1	Adipocyte enhancer-binding protein 1	Adipocyte proliferation, enhanced macrophage inflammatory responsiveness	Orphan
AMPD1	3	AMP deaminase 1	Energy metabolism	Orphan
CD14	15	Monocyte differentiation antigen CD14	Immune response	Orphan
CEACAM21	21	Carcinoembryonic Antigen Related Cell Adhesion Molecule 21	Immune response	Orphan
CLDN10	22	Claudin-10	Cell adhesion	Orphan
CPNE5	24	Copine-5	Melanocytes formation	Orphan
CTR	27	Calcitonin receptor	Receptor for calcitonin	Orphan
C12orf63	28	Cilia- and flagella-associated protein 54	Cilia and flagella assembly	Orphan
C8orf80	29	Nuclear GTPase, Germinal Center Associated	Genome stability	Orphan
DERL3	31	Derlin-3	Endoplasmic reticulum stress-induced pre-emptive quality control	Orphan
DLC1	32	Rho GTPase-activating protein 7	Cell proliferation and migration	Orphan
DPEP1	33	Dipeptidase 1	Immuno-inflammatory process	Orphan
FAM46C	36	Nucleotidyl transferase FAM46C	RNA polymerization	Orphan
FAM92B	37	Protein FAM92B	Ciliogenesis	Orphan
FCRL2	39	Fc receptor-like protein 2	Immune response, B-cells tumorigenesis	Orphan
FCRL5	40	Fc receptor-like protein 5	Immune response	Orphan
FLCN	42	Folliculin	Tumor suppression	Orphan
GALNT12	44	Polypeptide N-acetylgalactosaminyl transferase 12	Oligosaccharide biosynthesis	Orphan
GPR114	45	Adhesion G-protein coupled receptor G5	Cell signaling	Orphan
KCNA3	64	Potassium voltage-gated channel subfamily A member 3	Mediates the voltage-dependent potassium ion permeability of excitable membranes	Orphan
KCNN3	65	Small conductance calcium-activated potassium channel protein 3	Forms a voltage-independent potassium channel activated by intracellular calcium	Orphan
KLHL6	66	Kelch-like protein 6	Immune response	Orphan
LAX1	67	Lymphocyte trans membrane adapter 1	Immune response	Orphan
LBP	68	Lipopolysaccharide-binding protein	Immune response	Orphan
LGALS7	69	Galectin-7	Cell growth control	Orphan
LILRA3	70	Leukocyte Immunoglobulin Like Receptor A3	Immune response	Orphan
LY9	71	T-lymphocyte surface antigen Ly-9	Immune response	Orphan
LRMP	72	Lymphoid-restricted membrane protein	Immune response	Orphan
MCC	75	Colorectal mutant cancer protein	Tumor suppression	Orphan
MEI1	76	Meiosis inhibitor protein 1	Meiosis	Orphan
MLH3	78	DNA mismatch repair protein Mlh3	DNA repair	Orphan
MMP12	84	Macrophage metalloelastase	Tissue remodeling	Orphan
MUTYH	88	Adenine DNA glycosylase	DNA repair	Orphan
ODC1	93	Ornithine decarboxylase	DNA replication, cell proliferation, and apoptosis	Orphan
PDGFRL	95	Platelet-derived growth factor receptor-like protein	Associated with colorectal cancer and other malignancies	Orphan
PIM2	98	Serine/threonine-protein kinase pim-2	Cell proliferation, cell survival	Orphan
PNOC	107	Prepronociceptin	Nociception, neuronal development	Orphan
PTPN12	105	Tyrosine-protein phosphatase non-receptor type 12	Cell signaling	Orphan
PTPRJ	106	Receptor-type tyrosine-protein phosphatase eta	Cell proliferation and differentiation, cell adhesion and migration, platelet activation, and thrombosis	Orphan
P2RX1	107	P2X purinoceptor 1	Synaptic transmission	Orphan
RAD54B	108	DNA repair and recombination protein RAD54B	DNA repair	Orphan
RPS11	110	Ribosomal protein S11	40S sub-unit ribosomal protein	Orphan
SAA1	111	Serumamyloid A-1 protein	Inflammation	Orphan
SIRT2	112	NAD-dependent protein deacetylase sirtuin-2	Cell cycle regulation	Orphan
SLAMF7	113	SLAM family member 7	Immune response	Orphan
SLC17A9	114	Solute carrier family 17 member 9	ATP storage and exocytosis	Orphan
SPAG	118	RNA polymerase II-associated protein 3	RNA polymerization	Orphan
SSR4	119	Translocon-associated protein subunit delta	Retention of ER resident proteins regulation	Orphan
STK11	120	Serine/threonine-protein kinase STK11	Tumor suppression	Orphan
ST6GAL1	121	Beta-galactoside alpha-2,6-sialyltransferase 1	Transfers sialic acid from CMP-sialic acid to galactose-containing acceptor substrates	Orphan
TAS1R3	122	Taste receptor type 1 member 3	Umami taste stimulus response	Orphan
TMEM156	129	Transmembrane protein 156	Transmembrane protein	Orphan
TNFa	130	Tumor necrosis factor	Cell proliferation and differentiation, tumor cells death	Orphan
